# Disparities in Early Transitions to Obesity in Contemporary Multi-Ethnic U.S. Populations

**DOI:** 10.1371/journal.pone.0158025

**Published:** 2016-06-27

**Authors:** Christy L. Avery, Katelyn M. Holliday, Sujatro Chakladar, Joseph C. Engeda, Shakia T. Hardy, Jared P. Reis, Pamela J. Schreiner, Christina M. Shay, Martha L. Daviglus, Gerardo Heiss, Dan Yu Lin, Donglin Zeng

**Affiliations:** 1 Department of Epidemiology, the University of North Carolina at Chapel Hill, Chapel Hill, North Carolina, United States of America; 2 Department of Biostatistics, the University of North Carolina at Chapel Hill, Chapel Hill, North Carolina, United States of America; 3 Epidemiology Branch, Population and Prevention Sciences Program, Division of Cardiovascular Sciences, National Heart, Lung and Blood Institute, Bethesda, Maryland, United States of America; 4 Division of Epidemiology & Community Health, University of Minnesota, Minneapolis, Minnesota, United States of America; 5 Department of Nutrition, the University of North Carolina at Chapel Hill, Chapel Hill, North Carolina, United States of America; 6 Department of Medicine Institute for Minority Health Research, University of Illinois at Chicago, Chicago, Illinois, United States of America; Morehouse School of Medicine, UNITED STATES

## Abstract

**Background:**

Few studies have examined weight transitions in contemporary multi-ethnic populations spanning early childhood through adulthood despite the ability of such research to inform obesity prevention, control, and disparities reduction.

**Methods and Results:**

We characterized the ages at which African American, Caucasian, and Mexican American populations transitioned to overweight and obesity using contemporary and nationally representative cross-sectional National Health and Nutrition Examination Survey data (n = 21,220; aged 2–80 years). Age-, sex-, and race/ethnic-specific one-year net transition probabilities between body mass index-classified normal weight, overweight, and obesity were estimated using calibrated and validated Markov-type models that accommodated complex sampling. At age two, the obesity prevalence ranged from 7.3% in Caucasian males to 16.1% in Mexican American males. For all populations, estimated one-year overweight to obesity net transition probabilities peaked at age two and were highest for Mexican American males and African American females, for whom a net 12.3% (95% CI: 7.6%-17.0%) and 11.9% (95% CI: 8.5%-15.3%) of the overweight populations transitioned to obesity by age three, respectively. However, extrapolation to the 2010 U.S. population demonstrated that Mexican American males were the only population for whom net increases in obesity peaked during early childhood; age-specific net increases in obesity were approximately constant through the second decade of life for African Americans and Mexican American females and peaked at age 20 for Caucasians.

**Conclusions:**

African American and Mexican American populations shoulder elevated rates of many obesity-associated chronic diseases and disparities in early transitions to obesity could further increase these inequalities if left unaddressed.

## Introduction

An estimated 69% of American adults are overweight or obese [1], fueling a public health crisis of enormous financial cost [2,3] that has the potential to reverse gains in health and life expectancy achieved over the past century [4]. The prevalence of overweight and obesity has increased in all U.S. race/ethnic groups, with elevated burdens carried by minority populations, including African Americans and Mexican Americans [1]. African Americans and Mexican Americans also shoulder elevated rates of overweight- and obesity-associated chronic diseases including diabetes [5], stroke [6,7], coronary heart disease [8,9], and post-menopausal breast [10], prostate [11], and colorectal cancer [12], among others [13–16], when compared to Caucasians. Alarmingly, the burden of overweight and obesity in minority communities may maintain, or even increase, these persistent health disparities over the coming decades.

U.S. minority groups not only bear elevated burdens of overweight and obesity, but also may transition away from normal weight at younger ages when compared to Caucasian populations. For example, nationally representative estimates from 1996–2001 suggested that the incidence of obesity during the transition period between adolescence and adulthood was highest for African American females and Hispanic females [17]. Disparities in the burden of overweight and obesity, especially in young populations, are particularly concerning given research demonstrating difficulties reattaining normal body weight once classified as overweight or obese [17] as well as adverse effects from the length of time populations were overweight or obese [18,19]. However, no contemporary study to the best of our knowledge has characterized the ages at which multi-ethnic U.S. populations transition between normal weight, overweight, and obesity in populations spanning early childhood through late adulthood. Such research is needed given strong secular trends in overweight and obesity over the past three decades [20] that may limit the generalizability of earlier studies to present day, historic obesity disparities [1] supporting studies with large numbers of minority populations, and the ability of research spanning early childhood through late adulthood to broadly inform obesity prevention and control efforts. We therefore leveraged cross-sectional data from n = 21,220 participants of African American, Caucasian, and Mexican American descent from a large contemporary national sample and calibrated and validated novel Markov-type models to examine race/ethnic-, sex-, and age-specific (2–80 years) net transitions between normal weight, overweight, and obesity.

## Materials and Methods

### Ethics statement

The Institutional Review Board at the University of North Carolina approved the study protocol. This study was conducted according to the principles expressed in the Declaration of Helsinki.

### Study population

We characterized the ages at which African American, Caucasian, and Mexican populations transitioned between normal weight, overweight, and obesity using cross-sectional data from the National Health and Nutrition Examination Survey (NHANES) [21]. NHANES is a complex, multistage probability sample of the U.S. population (aged 0–80+ years; participants ≥80 years of age were collapsed into age 80 by NHANES investigators) conducted by the National Center for Health Statistics that measured demographic, dietary, and health-related traits [21]. For this study, we used data from three continuous NHANES population cross-sections (i.e. 2007–08, 2009–10, and 2011–12) and evaluated non-Hispanic Caucasian (n = 10,224), non-Hispanic African American (n = 6,069), and Mexican American (n = 4,927) participants 2–80 years of age. We excluded non-Hispanic Asian participants who were available as of the 2011–12 population cross-section as well as participants designated as “Other Hispanic” or “Other Race—Including Multi-Racial” due to small sample sizes and, for the latter, uncertainty regarding the underlying source population. The study was approved by local institutional review boards and all participants gave written informed consent.

### Measurement and classification of weight

Weight was classified according to the previously published weight cardiovascular health metric [22]. For adults ≥20 years of age, BMI was measured as kg/m^2^ using standard equipment and categorized as normal weight (BMI <25 kg/m^2^), overweight (BMI 25–29.99 kg/m^2^), or obese (BMI ≥30 kg/m^2^). Less than 1% of each race/ethnic and sex-specific population was classified as underweight (BMI < 18.5). For participants 2–19 years of age, BMI was characterized as normal weight (<85^th^ percentile), overweight (85^th^-95^th^ percentile), or obese (>95^th^ percentile) using sex-specific growth curves developed by the Centers for Disease Control and Prevention [23]. Participants who were pregnant, recently pregnant, who had a limb amputation, or were missing variables to calculate BMI were excluded. Children 0–1 years of age also were excluded from the cardiovascular health BMI guidelines and were therefore not considered herein.

### Statistical analysis

#### Estimation of net transition probabilities

We characterized the ages at which African American, Caucasian, and Mexican American populations transitioned between normal weight, overweight, and obesity using calibrated and validated Markov-type models that estimated net transition probabilities from cross-sectional data [24]. For example, in a longitudinal study of 100 normal weight participants aged 18 years, if 10 participants transitioned from normal weight to overweight by age 19 and three participants transitioned from overweight to normal weight by age 19, the normal weight to overweight net transition would be seven participants; dividing by the number of normal weight participants at age 18 (n = 100) would yield the net transition probability, here 7%. The overweight to normal weight net transition is 0 because fewer participants moved from overweight to normal weight than from normal weight to overweight. Under the assumption that the age-specific net transitions probabilities remained approximately stable across time (see below and S1 Appendix), we leveraged cross-sectional data to estimate net transition probabilities; estimation of the number of participants transitioning from normal weight to overweight and overweight to normal weight (i.e. individual transition probabilities) would require longitudinal data spanning early childhood to late adulthood in multi-ethnic studies, which is unavailable, or if available, likely conducted in prior decades and therefore potentially outdated due to strong overweight and obesity secular trends [20].

**Fig 1:**
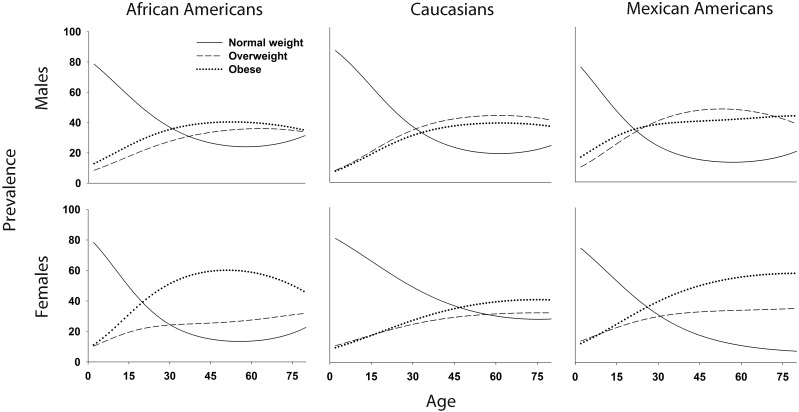
Smoothed age (2–80 years)-, race/ethnic-, and sex-specific prevalence proportions of normal weight (solid line), overweight (dashed line), and obesity (dotted line) estimated in n = 21,220 NHANES participants.

Estimation of net transition probabilities from cross-sectional data required three steps. First, we estimated the age-, sex-, and race/ethnic-specific prevalence of normal weight, overweight, and obesity using a multinomial logit model [25,26] and P-spline smoothing [27] that accommodated using cluster sampling methods. Although weight was categorized using percentiles for participants 2–19 years of age and standard BMI cut-points for participants ≥ 20 years of age, we observed no evidence of discontinuities by race/ethnicity or sex across the two age groups and therefore presented smoothed results across 2–80 years of age. Next, we used a series of simplex algorithms from linear programming theory to estimate age-, sex-, and race/ethnic-specific net transition probabilities (Fig 1). Finally, bootstrapping was used to estimate 95% confidence intervals, where the age-specific prevalence was simulated from its asymptotic distribution and net transition probabilities were computed for each simulated prevalence [24].

Population extrapolations of the one-year age- and sex-specific net number of non-institutionalized African Americans, Caucasians, and Mexican American males and females transitioning from normal weight to overweight (and overweight to obesity) were calculated by multiplying the age (2–80), sex-, and race/ethnic-specific normal weight to overweight (overweight to obesity) net transition probabilities by the prevalence of normal weight (overweight) and the race/ethnic-, sex- and age-specific 2010 civilian noninstitutionalized population size. Statistical analyses were performed using SAS (Cary, NC), STATA (College Station, TX), and R (Vienna, Austria).

#### Calibration of net transition probabilities

Estimation of net transition probabilities required the specification of a cost constant, *c*_*ij*_. The cost constant was used to impose an “economy of movement,” where remaining in the current weight category was always cheapest (i.e. “zero-step”) and two “one-step” movements (e.g. normal weight to overweight) were always cheaper than one “two-step” movement (normal weight to obesity). Conceptually, the cost constant reflected our belief that movement from normal weight to obesity (or vice-versa) in one year was unlikely; instead, staying in the same weight category or a one-step movement (i.e. from normal weight to overweight) were more plausible. Results of our calibration study using longitudinal Coronary Artery Risk Development in Young Adults (CARDIA) study data [28] suggested that the cost constants of 0, 6, and 17 were optimal for describing zero-, one-, and two-step movements between weight categories (S1 Appendix).

#### Validation of net transition probabilities

Net transition probabilities can be validly estimated from cross-sectional data under the assumption that the transitions remain stable across time [24]. To evaluate this assumption, we conducted a series of simulations that evaluated the ability of earlier NHANES population cross-sections to predict BMI prevalence proportions estimated in later cross-sections. Our results suggested stability in weight transitions between 2007–12 (S1 Fig), thereby validating the use of net transition probabilities to examine one-year weight net transitions.

## Results

A total of 21,220 participants (28.6% African American; 48.2% Caucasian; 23.2% Mexican American) aged 2–80 were available for analysis (Table 1). African Americans (median age range: 30–35 years) and Mexican Americans (median age range: 26–27 years) were on average younger than Caucasians (median age range: 40–43 years) and there were approximately equal proportions of males and females across each race/ethnicity (weighted proportion of females range: 49.6%-52.8%). At age two, the prevalence of normal weight ranged from 73.8%—85.0%, with the lowest prevalence observed in Mexican American females and the highest in Caucasian males (Fig 1). Mexican American males had the highest prevalence of obesity at age two (16.1%).

**Table 1:** Race/ethnic- and sex-specific demographics for n = 21,220 NHANES (2007–12) participants 2–80 years of age used to characterize the age-specific net probability of transitioning between normal weight, overweight, and obesity. BMI, body mass index; N, unweighted number; IQR, interquartile range.

Characteristic	African American		Caucasians		Mexican Americans	
	Females	Males	Females	Males	Females	Males
N.	3,026	3,043	4,998	5,226	2,417	2,510
Median age (IQR)	35 (17, 52)	30 (16, 49)	43 (23, 59)	40 (21, 56)	26 (12, 43)	27 (13, 41)
Weight category						
% Normal weight	31.3	42.5	44.7	36.7	38.9	33.8
% Overweight	22.9	25.5	26.1	33.8	26.2	33.2
% Obese	45.7	31.9	29.2	29.5	34.9	33.0
Median BMI percentile (IQR), children 2–19	74.0 (44.7, 93.8)	71.0 (41.4, 92.8)	65.0 (37.6, 87.6)	64.8 (35.9, 88.2)	73.3 (45.3, 92.3)	77.2 (48.0, 94.7)
Median BMI (IQR), adults 20–80	31.0 (26.1, 36.6)	27.9 (23.9, 32.7)	26.9 (23.2, 32.0)	27.8 (24.8, 31.4)	29.2 (25.2, 33.9)	28.5 (25.8, 31.9)

**Fig 2:**
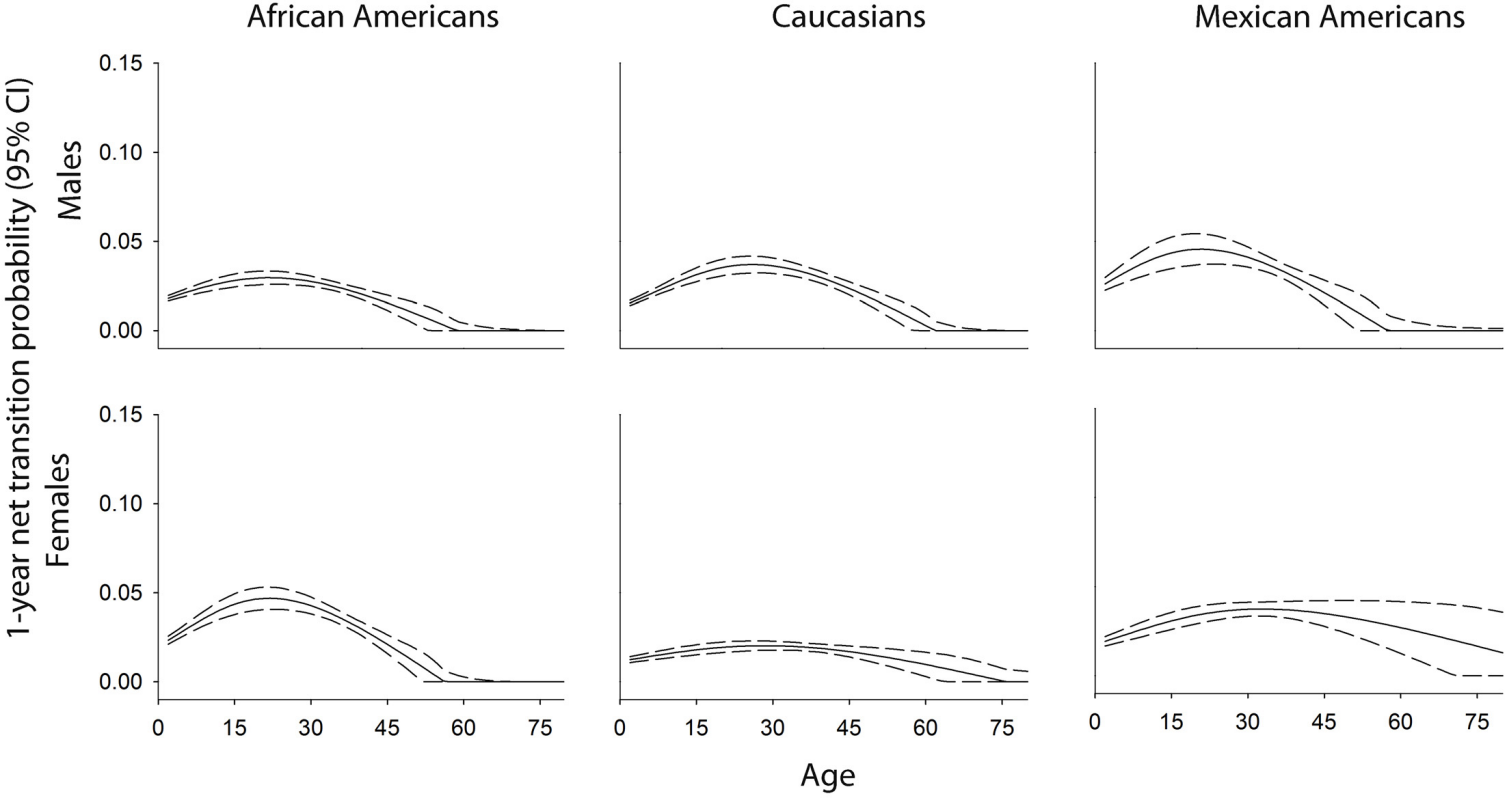
Age (2–80 years) race/ethnic-, and sex-specific normal weight–to- overweight net transition probabilities estimated in n = 21,220 NHANES participants.

**Fig 3:**
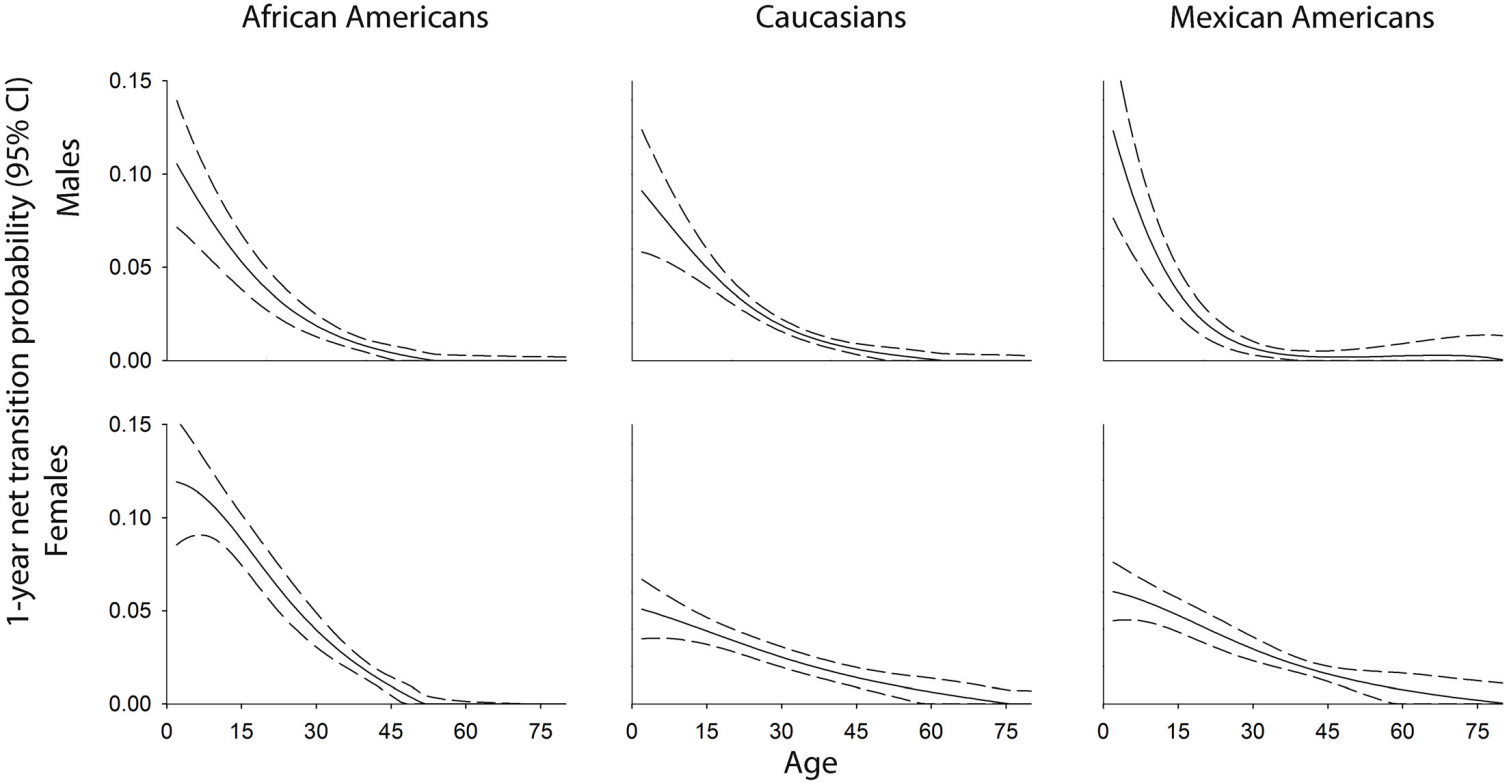
Age (2–80 years)-, race/ethnic-, and sex-specific overweight-to-obesity net transition probabilities estimated in n = 21,220 NHANES participants.

Estimated net transition probability patterns were generally comparable by sex and race/ethnicity (Figs 2 and 3; S1 Table), although heterogeneity was observed for both the magnitude of net transitions as well as the ages at which net transition probabilities peaked. The highest normal weight to overweight net transition probabilities were estimated for African American females 22 years of age, for whom a net 4.7% [95% confidence interval (CI): 4.1%, 5.3%] of the normal weight population transitioned to overweight by age 23 (Fig 2). Net normal weight to overweight net transition probabilities also peaked during the 20s for the remaining populations, with the exception of Mexican American females, for whom the net transition probability to overweight [net transition probability = 3.7% (95% CI: 3.4, 4.1)] peaked at 33 years of age. Mexican American females also were the only population for whom normal weight to overweight net transition probabilities were consistently favored from two to 80 years of age; for the remaining populations, larger proportions of the population moved from normal weight to overweight than from overweight to normal weight until approximately 62 years of age (race/ethnic and sex-specific range: 57–76 years) when overweight to normal weight net transitions were favored, represented by net transition probabilities = 0.

Unlike normal weight to overweight net transitions probabilities that peaked in early adulthood for all populations, estimated one-year net transition probabilities from overweight to obesity peaked at age two for all populations and were highest for Mexican American males, where a net 12.3% (95% CI: 7.6%, 17.0%) of the overweight population transitioned to obesity by age three. Elevated net transition probabilities at age two also were estimated for African American females [net transition probability = 11.9% (95% CI: 8.5%, 15.3%)], African American males [net transition probability = 10.5% (95% CI: 7.1%, 13.9%)], and Caucasian males [net transition probability = 9.1% (95% CI: 5.8, 12.4)], considerably higher than net transition probabilities estimated for Caucasian females [net transition probability = 5.1% (95% CI: 3.5%, 6.7%)] and Mexican American females [net transition probability = 6.0% (95% CI: 4.5%, 7.6%)]. In addition to having the highest estimated overweight to obesity net transition probability at age two, Mexican American males also showed the steepest declines in overweight to obesity net transition probabilities, which declined to approximately 1% at 27 years of age (Fig 3,S1 Table). In contrast, overweight to obesity net transition probabilities for Caucasian females and Mexican American females remained elevated through the fifth decade of life.

**Fig 4:**
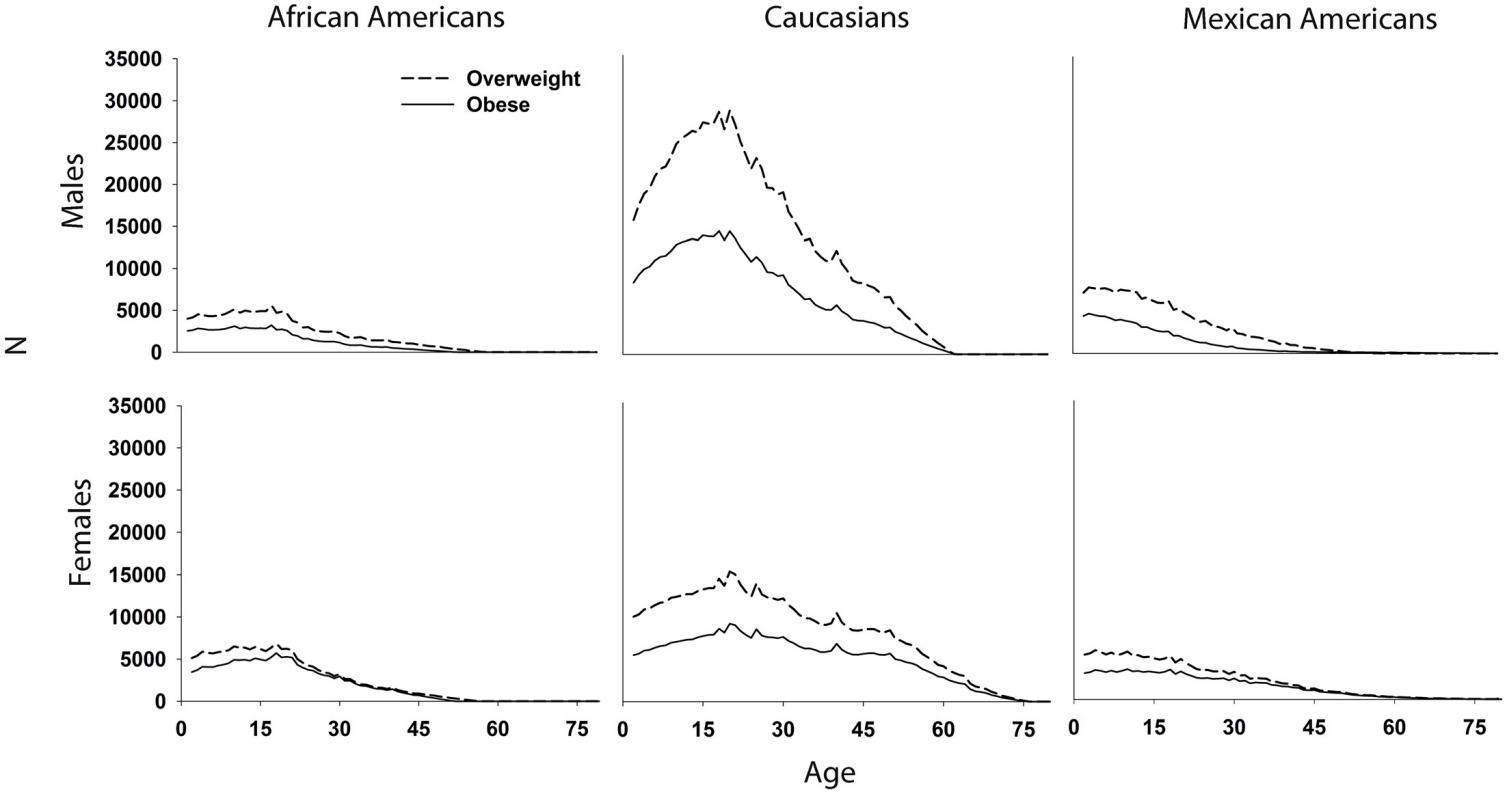
One-year age-specific population extrapolations of the net number of non-institutionalized African American, Caucasian, and Mexican American males and females 2–80 years of age transitioning to overweight and obesity.

Extrapolation to the African American, Caucasian, and Mexican American 2010 civilian noninstitutionalized U.S. population two to 80 years of age demonstrated disparities in the ages associated with the largest net increases in overweight and obesity (Fig 4). Mexican American males were the only population for whom the largest net increases in obesity occurred during early childhood. For African American males and females and Mexican American females, age-specific net increases in obesity were approximately constant from early childhood through the second decade of life, whereas net increases in obesity peaked at age 20 for Caucasian males and females. Among African American males, Caucasian males and females, and Mexican American males, net increases in overweight always exceeded net increases in obesity. However, for African American females and Mexican American females, net increases in overweight and obesity converged by 30 and 45 years of age, respectively.

## Discussion

Our multi-ethnic study spanning early childhood to late adulthood demonstrated that obesity disparities were apparent by two years of age and were sustained through late adulthood. Elevated net transition probabilities to overweight and obesity in childhood and adolescence exacerbated these obesity disparities, particularly for African American females and Mexican American males and females. Heterogeneity in the ages at which the largest net increases in overweight and obesity occurred also was observed and should be considered when developing and implementing overweight and obesity prevention and control programs.

Very few studies have evaluated transitions to overweight and obesity in large, multi-ethnic populations that span early childhood to late adulthood. Instead, available studies typically span specific life epochs. For example, the Early Childhood Longitudinal Study (ECLS) demonstrated that obesity incidence peaked at approximately six years of age for overweight school-age children, but remained approximately constant among the total cohort between five and 14 years of age [29]. The adolescent to early adulthood obesity transition was examined by National Longitudinal Study of Adolescent to Adult Health (Add Health) investigators, who reported that African American and Hispanic females as well as older adolescents had the highest incidence of obesity [17]. The lack of studies spanning these two (and other) critical age epochs underscores the difficulties stakeholders face when trying to understand weight transitions across the life course, as it is unclear whether results from studies like ECLS and Add Health can be pieced together with studies of adult populations [30] to provide accurate and widely generalizable inferences across broad age epochs. Existing studies of obesity transitions also are limited by modest numbers of minority participants and were conducted one or two decades ago despite obesity secular trends, potentially limiting their ability to inform contemporary race/ethnic- and sex-specific weight transition patterns.

Given the challenges described above, it is not surprising that contemporary prevalence estimates in multi-ethnic populations currently form the basis by which the burden of overweight and obesity in U.S. populations is monitored [1,31,32]. However, we identified several important and novel features by extending these prevalence estimates to evaluate net transitions. First, for all populations, overweight to obesity net transition probabilities peaked at age two, although Mexican American males were the only population for whom the largest net increases in obesity occurred during early childhood. These seemingly incongruent results reflect the interplay between population-specific factors including the magnitude of net transition probabilities, patterns of change by age, and the prevalence of normal weight, overweight, and obesity, therefore providing several avenues to target for obesity prevention, control, and, ultimately, disparities reduction. Interestingly, the only behavioral intervention endorsed by the Community Preventive Services Task Force (CPSTF) for obesity prevention and control in children—reducing screen time—is recommended for children 13 years of age and younger [33] and therefore may help alleviate obesity disparities given population extrapolations suggesting larger proportional increases in obesity from early childhood throughout 13 years of age in African Americans and Mexican Americans when compared to Caucasians. However, net transitions to overweight and obesity remain elevated for all populations through later adolescence and early adulthood, necessitating additional research to prevent or reverse obesity during these critical age epochs.

The intermediate role of overweight also was masked by previous studies of prevalence. For example, among African American females, the net numbers transitioning to overweight were always equal to or higher than then net numbers transitioning to obesity. However, the prevalence of obesity was higher than the prevalence of overweight across all ages. These seemingly incongruent observations reflected the intermediate role of overweight, as large normal weight to overweight net transitions were offset by concurrently large overweight to obesity net transitions that blunted increases in the prevalence of overweight. It remains important to understand the extent to which the previously reported stable prevalence of overweight over the past five decades [1,20] as well as any future overweight trends, reflect changes in net transitions from normal weight to overweight, changes in net transitions from overweight to obesity, or a combination of the two.

Heterogeneity in overweight and obesity transitions across the life course also merits consideration when designing and implementing obesity prevention and control programs, as failure to consider such heterogeneity could potentially exacerbate obesity disparities. For example, obesity control interventions, i.e. interventions that reduce obesity prevalence, may be a more effective means of reducing the obesity burden than obesity prevention interventions in Mexican American adult males, for whom the greatest net population shifts to obesity occurred by the mid-20s. Examples of evidence-based obesity control interventions in adult populations include worksite programs and technology-supported multicomponent coaching to reduce weight [33,34]. In contrast, few evidence-based programs have been recommended for adult obesity prevention, although overweight to obesity net transition probabilities generally remained elevated through adulthood, particularly for Mexican American females. However, few studies have simultaneously evaluated obesity prevention and control interventions in multi-ethnic populations spanning childhood to late adulthood to understand the implications of different evidence-based and hypothetical prevention and control strategies.

Despite numerous strengths, this study has several limitations that deserve consideration. First, our study was limited to African Americans, Caucasians, and Mexican Americans, as large, contemporary studies of other multi-ethnic populations spanning childhood to late adulthood were unavailable. However, Mexican Americans are the largest Hispanic-origin population in the U.S., accounting for 64% of the 2012 U.S. Hispanic population [35], and when combined with African Americans and Caucasians compose approximately 86% of the U.S. population [36]. Second, we calibrated net transition probabilities in a biracial (African American and Caucasian) population spanning late adolescence through middle adulthood, although we report net transitions from early childhood through late adulthood, although our study included African American, Caucasian, and Mexican American participants 2–80 years of age. Our extensive calibration study demonstrated that changing the numeric value of cost constants produced extremely small changes in estimated net transition probabilities, as previously demonstrated [24], suggesting that differences in cost constants calculated within younger or older populations would have minimal effects on study findings. However, we did not evaluate the influence of other annual “economy of movement” patterns that could potentially have larger effects on estimated net transition probabilities, e.g. scenarios where movement out of a current weight category was more likely than remaining in the current weight category over one year. Yet, our application of the originally proposed “economy of movement” scenarios is supported by longitudinal studies of children [37] and adults [38], which reported modest changes in weight and thus weight categories over the short time periods evaluated herein, thereby lending additional support to our chosen approach. Third, we did not incorporate any contextual factors available in NHANES, including socioeconomic status, food security, and diet. Studies evaluating whether these and other measures modify the observed net transition probabilities are therefore warranted. Finally, our validation study only supported estimation of one-year net transition probabilities; estimation of five-year, 10-year, or 20-year net (or individual) transitions would require longitudinal data. Nonetheless, one-year net transition probabilities capture important and contemporary population-level dynamics for immediate clinical and public health action, can be calculated for a wide range of population health metrics (e.g. hypertension, physical activity, and diabetes), and may be particularly useful in resource poor settings where longitudinal studies are infeasible.

By late adolescence, large proportions of the African American, Caucasian, and Mexican American populations have transitioned from normal weight. Each population showed distinct patterning in the ages at which peak population shifts to overweight and obesity occurred, with earlier and sustained transitions from normal weight observed in African Americans and Mexican Americans. U.S. minorities already shoulder elevated rates of many obesity-associated chronic diseases and the early transitions from normal weight may further increase these disparities if left unaddressed.

## Supporting Information

S1 Appendix Appendix describing analytic methods in additional detail and results of the calibration and validation studies. (DOCX)

S1 Fig Simulation results examining the stability of weight category transitions by age (2–80 years) in n = 21,220 African American, Caucasian, and Mexican American male and female NHANES participants, 2007–2012. The stability of weight category transitions across time is assessed by the ability of net transition probabilities estimated in the 2007–2008 NHANES population cross-sections to predict the prevalence of normal weight, overweight, and obesity in the 2009–2010 (panels A, B, and C) and 2011–2012 (panels D, E, and F) independent NHANES population cross-sections.
(TIF)

S1 Table Selected age (2–80 years)-, race/ethnic (African American, Caucasian, and Mexican American)-, and sex-specific net transition probabilities between normal weight and overweight and overweight and obesity estimated in n = 21,220 National Health and Nutrition Examination Survey (NHANES) participants. (DOCX)
