# Supplementary material for: Disparities in Early Transitions to Obesity in Contemporary Multi-Ethnic U.S. Populations
Source: PLoS One. 2016 Jun 27;11(6):e0158025. doi: 10.1371/journal.pone.0158025 (PMC4922630; doi:10.1371/journal.pone.0158025)
Supplement: S1 Appendix — (DOCX) [file pone.0158025.s001.docx]

**Supporting Information**

**Estimation of Net Transition Probabilities**

Our approach to estimating net transition probabilities was built upon a foundation established by operations research, allowing us to view the estimation of age-specific net transition probabilities between weight categories as a transportation problem Briefly, the transportation problem was conceptualized by specifying supplies, demands, shipping costs, decision variables, and an objective function. The supplies were interpreted as smoothed prevalence proportions $\hat{\pi_{i}}(a-1)$, where *a* represented age and *i* indexed the weight category at age *a-1*, the demands were the prevalence proportions one year later $\hat{\pi_{j}}(a)$, *j* indexed the weight category at age *a,* the cost constants were specified beforehand (*c_ij_,* see below), the decision variables were the net transitions ($\tau_{ij}^{'}(a)$, to be calculated) and the objective function *J* kept track of the total transportation costs between weight categories at age *a-1* to weight categories at age *a*. The objective function *J* was then minimized: $min J= \sum_{i=1}^{3} \sum_{j=1}^{3} c_{ij}\tau_{ij}^{'}(a)$, subject to the conditions $\sum_{j=1}^{3} \tau_{ij}^{'}\left( a \right)=\hat{\pi_{i}}\left( a-1 \right), \sum_{i=1}^{3} \tau_{ij}^{'}\left( a \right)=\hat{\pi_{j}}(a)$ and $\tau_{ij}^{'}\left( a \right)\geq0$. The conditions ensured that the total flow from weight category *i* represented the supply of that level, that the total flow into weight category *j* was equal to the demand of that level, the overall supply always equalled the overall demand, and that no transitions were negative. The net transition probabilities $p_{ij}^{'}(a)$ were then estimated as $p_{ij}^{'}\left( a \right)=\tau_{ij}^{'}(a)/\hat{\pi_{i}}(a-1)$.

Calibration of Net Transition Probabilities

**Overview**

Estimation of net transition probabilities requires the specification of a cost constant, *c_ij_*, yet no study has attempted to calibrated cost constraints that describe movement within and between weight categories using longitudinal data.

**Approach**

Initially we assigned zero costs to remaining within the same weight category, a cost of one unit for moving one level up or down (e.g. normal weight to overweight), and a cost of three units for moving two levels up or two levels down (e.g. normal weight to obesity), as previously recommended [[1](#_ENREF_1)]. This initial approach was then contrasted with cost constraints calculated using longitudinal data. Specifically, to calibrate cost constraints, we used longitudinal data from the Coronary Artery Risk Development in Young Adults (CARDIA) study, a multi-center, population-based study of the evolution of coronary heart disease risk beginning in young adulthood [[2](#_ENREF_2)]. CARDIA investigators recruited n=5,115 African American (51.5%) and Caucasians (48.5%) males and females aged 18-30 at study baseline (1985-1986) from four US communities. BMI was measured at eight visits over 25 years and CARDIA participants contributed a mean of 6.3 repeat BMI measures. Although CARDIA data are not ideal to describe contemporary weight transitions given the large secular changes in overweight and obesity that have occurred over the past three decades and the restriction to African American and Caucasians populations, they can inform on the likelihood of moving between weight categories.

To calibrate the cost constraints, we first estimated the age-specific cumulative probabilities for normal weight (0), overweight (1), and obesity (2) as $\theta_{0}, \theta_{1}$ and$\theta_{2}$=1, respectively, using a cumulative logistic mixed effects model.

$$\theta_{0}(age,b)=\frac{\exp(\alpha_{0}+\beta_{1}\times age+ \beta_{2}\times age^{2}+\gamma_{1}\times I(race=white)+\gamma_{2}\times I(sex=female)+\gamma_{3}\times I(race=white,sex=female)+b)}{1+ \exp(\alpha_{0}+\beta_{1}\times age+ \beta_{2}\times age^{2}+\gamma_{1}\times I(race=white)+\gamma_{2}\times I(sex=female)+\gamma_{3}\times I(race=white,sex=female)+b) }$$

$$\theta_{1}(age,b)=\frac{\exp(\alpha_{1}+\beta_{1}\times age+ \beta_{2}\times age^{2}+\gamma_{1}\times I(race=white)+\gamma_{2}\times I(sex=female)+\gamma_{3}\times I(race=white,sex=female)+b) }{1+ \exp(\alpha_{1}+\beta_{1}\times age+ \beta_{2}\times age^{2}+\gamma_{1}\times I(race=white)+\gamma_{2}\times I(sex=female)+\gamma_{3}\times I(race=white,sex=female)+b )}$$

Here, $\alpha_{0}$. $\alpha_{1}$, $\beta_{1}$, $\beta_{2}$, $\gamma_{1}$, $\gamma_{2}$ and $\gamma_{3}$ are fixed effects and $b$ are random effects assumed to follow a normal distribution with mean zero. The probabilities of normal weight, overweight, and obesity given $b$ are respectively:

$$\pi_{0}= \theta_{0},\pi_{1}= \theta_{1}-\theta_{0},\pi_{2}= 1-\theta_{1}.$$

The prevalence for weight category $j$ at age $(a-1)$ is

$$\int_{-\infty}^{\infty} \pi_{j}\left(a-1,b\right)\times\frac{1}{\sqrt{2\pi}\sigma}\exp\left(-\frac{b^{2}}{2\sigma^{2}}\right) db$$

and the net transition from group $j$ to group $i$ for $j\neq i$ is

$$max(\int_{-\infty}^{\infty} \pi_{i}(a,b)\times\pi_{j}\left(a-1,b\right)\times\frac{1}{\sqrt{2\pi}\sigma}\exp\left(-\frac{b^{2}}{2\sigma^{2}}\right) db$$

$$-\int_{-\infty}^{\infty} \pi_{j}(a,b)\times\pi_{i}\left(a-1,b\right)\times\frac{1}{\sqrt{2\pi}\sigma}\exp\left(-\frac{b^{2}}{2\sigma^{2}}\right) db,0)$$

For $i=j$, the net transition from group $i$ to $i$ at age $a$ is defined as

prevalence of group $i$ at age (a-1) - net transition from group $i$ to $k$.

The net transition probability at age $a$ from weight category $i$ to weight category $j$ is then defined as:

$$\frac{\text{net transition from i to j at age a}}{\text{Prevalence of group i at age a}}$$

The net transition probability is then obtained for each of the four race-sex groups and then averaged over the four groups.

We then used a numerical integration approach to calculate the integrals described in the above formulas [[3](#_ENREF_3)] that estimated net transition probabilities from the cross sectional model using the optimization algorithm approach for a given set of cost parameters and also net transition probabilities from the longitudinal model obtained by fitting the cumulative logistic mixed effects model. Calibration errors were calculated as net transition probability obtained from the cross sectional model - net transition probability obtained from the longitudinal model|.

The net transition probability obtained from the cross sectional model were based on the baseline value only. The cost parameters were searched in [0,20] with an increment of one, ensuring that the cost of remaining in the same group were less than that of transitioning to a different group and the cost of transitioning from category 0 to category 2 or category 2 to category 0 were larger than the sum of the other two cost parameters. The optimal cost parameters were then chosen as the parameters for which the calibration error was minimum.

*Results.* Our results showed that net transitions estimated using optimized cost constraints of 0, 8, and 17 calculated using longitudinal CARDIA data produced net transitions and standard errors that differed on average less than 0.01% from net transitions estimated using initial cost constraints (0, 1, and 3), suggesting little influence of cost constraint definition on the estimation of net transitions or associated standard errors.

**Validation of Net Transition Probabilities**

*Overview.* To evaluate the assumption that weight category transitions remained approximately stable across time, we compared observed weight category prevalence proportions calculated from the 2009-2010 and 2011-2012 NHANES data with estimated 2009-2010 and 2011-2012 weight category prevalence proportions calculated from net transition probabilities generated from 2007-2008 NHANES data. Overlap between observed and estimated weight category prevalence proportions in 2009-2010 and 2011-2012 would suggest that weight category transitions remained approximately stable across the time period of examination, here approximately four years.

**Approach**

First, we calculated age-specific weight category net transition probabilities using the 2007-2008 NHANES data in the race/ethnic- and sex-combined African American, Caucasian, and Mexican American sample using the approach described in the main paper.[[1](#_ENREF_1)] The 2007-2008 net transition probabilities were then used to estimate the prevalence of normal weight, overweight, and obesity two years (corresponding to the 2009-2010 NHANES data) and four years (corresponding to the 2011-2012 NHANES data) later. To estimate the expected age-specific prevalence of normal weight, overweight, and obesity in 2009-2010 from 2007-2008 data, we defined an age *a* population of $n_{a0}$ normal weight participants, $n_{a1}$ overweight participants, and $n_{a2}$ obese participants in 2007-2008, noting that the same approach was used to estimate the weight category prevalence proportions in 2011-2012 from 2007-2008 data. At age *a*, the two year net transition probability from weight category $i$ to weight category $j$ was $p_{aij}$ . The two year net transition probability was obtained by multiplying the one year net transition probability matrix with itself for each age. We then:

1. Estimated $x_{j}=(x_{j0}, x_{j1}, x_{j2})$, the expected number of participants in moving from weight category j to each weight category (including j) by 2009-2010 as $n_{aj}\times( p_{aj0}, p_{aj1} , p_{aj2} )$ for $j$ in $\{0,1,2\}$.
2. The proportion of participants in each weight category in 2009-2010 (indexed by *k*) is given as $=\frac{y_{ak}}{\sum_{i=0}^{2} y_{ai}}$ where $y_{ak}= \sum_{j=0}^{2} x_{jk}$.
3. Finally, we used locally weighted scatterplot smoothing (LOESS) to smooth the prevalence proportions across age.

**Results**

The results of the simulation are summarized in plots comparing the expected and observed weight category prevalence proportions; the first time point denotes 2007-2008, the second time point denotes 2009-2010, and third time point denotes 2011-2012. From the plots we can see that the curve of the smoothed expected and observed weight category proportions are highly concordant, suggesting that weight category transitions remained approximately stable across two and four years. Modest discrepancies between observed and expected estimates of normal weight in the 2011-2012 NHANES data are observed from middle to late adulthood (S1 Fig, panel D), although expected overweight (S1 Fig 1, panel E) and obesity (S1 Fig, panel F) prevalence estimates coincide with observed results, again suggesting that weight category transitions remained approximately stable across two and four years.

**Supplemental References**

1. Kassteele J, Hoogenveen RT, Engelfriet PM, Baal PH, Boshuizen HC (2012) Estimating net transition probabilities from cross-sectional data with application to risk factors in chronic disease modeling. Stat Med 31: 533-543.

2. Friedman GD, Cutter GR, Donahue RP, Hughes GH, Hulley SB, Jacobs DR Jr et al. (1988) CARDIA: study design, recruitment, and some characteristics of the examined subjects. J Clin Epidemiol 41: 1105-1116.

3. Golub GH, Welsch JH (1969) Calculation of Gauss Quadrature Rules. Mathematics of Computation 23: 221-230.
