# Supplementary figures and images for: Disparities in Early Transitions to Obesity in Contemporary Multi-Ethnic U.S. Populations
Source: PLoS One. 2016 Jun 27;11(6):e0158025. doi: 10.1371/journal.pone.0158025 (PMC4922630; doi:10.1371/journal.pone.0158025)

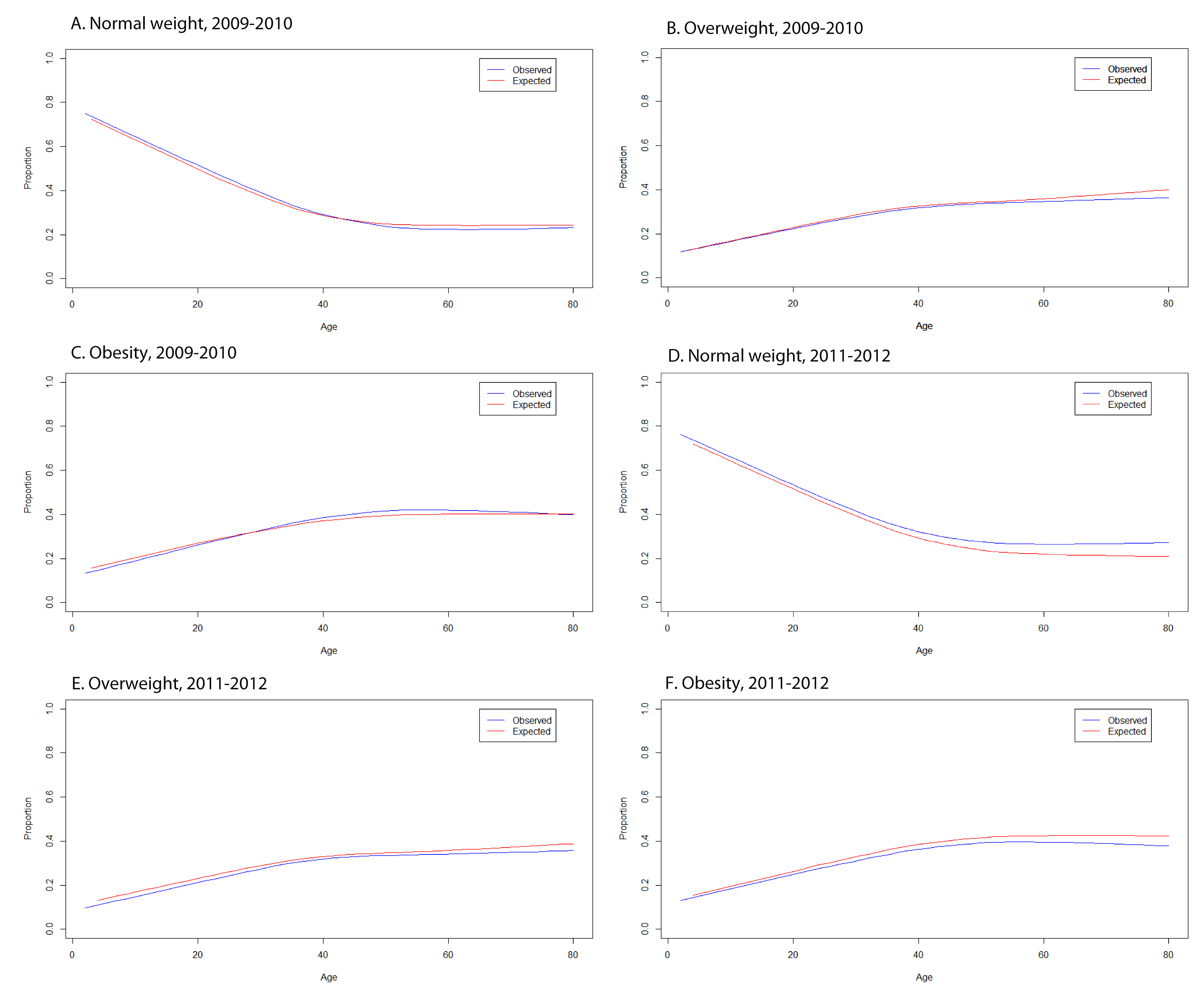

Supplement: S1 Fig — The stability of weight category transitions across time is assessed by the ability of net transition probabilities estimated in the 2007–2008 NHANES population cross-sections to predict the prevalence of normal weight, overweight, and obesity in the 2009–2010 (panels A, B, and C) and 2011–2012 (panels D, E, and F) independent NHANES population cross-sections. (TIF) [file pone.0158025.s002.tif]
