# Supplementary material for: Disparities in Early Transitions to Obesity in Contemporary Multi-Ethnic U.S. Populations
Source: PLoS One. 2016 Jun 27;11(6):e0158025. doi: 10.1371/journal.pone.0158025 (PMC4922630; doi:10.1371/journal.pone.0158025)
Supplement: S1 Table — (DOCX) [file pone.0158025.s003.docx]

|  |  | Estimated net transition probabilities (95% confidence interval) | | | | | |
| --- | --- | --- | --- | --- | --- | --- | --- |
|  |  | African Americans | | Caucasians | | Mexican Americans | |
| Transition | Age | Females | Males | Females | Males | Females | Males |
| Normal weight-overweight | 5 | 2.9 (2.6, 3.2) | 2.1 (1.9, 2.3) | 1.4 (1.2, 1.6) | 1.9 (1.7, 2.1) | 2.2 (1.9, 2.5) | 3.1 (2.6, 3.6) |
|  | 15 | 4.4 (3.8, 4.9) | 2.8 (2.4, 3.2) | 1.8 (1.5, 2.1) | 3.1 (2.7, 3.5) | 3.1 (2.6, 3.6) | 4.4 (3.5, 5.2) |
|  | 25 | 4.6 (4.0, 5.2) | 2.9 (2.6, 3.3) | 2.0 (1.7, 2.3) | 3.7 (3.2, 4.2) | 3.6 (3.2, 4.1) | 4.5 (3.7, 5.2) |
|  | 35 | 3.7 (3.3, 4.1) | 2.4 (2.2, 2.7) | 2.0 (1.7, 2.2) | 3.3 (3.0, 3.7) | 3.7 (3.3, 4.2) | 3.6 (3.1, 4.0) |
|  | 45 | 2.1 (1.6, 2.6) | 1.5 (1.1, 2.0) | 1.7 (1.4, 2.0) | 2.4 (2.0, 2.7) | 3.5 (2.8, 4.2) | 2.1 (1.4, 2.8) |
|  | 55 | 0.23 (0, 0.95) | 0.43 (0, 1.1) | 1.2 (0, 1.8) | 1.0 (0, 1.7) | 3.0 (1.8, 4.2) | 0.42 (0, 1.4) |
|  | 65 | 0 (0, 0) | 0 (0, 0) | 0.67 (0, 1.5) | 0 (0, 0.27) | 2.4 (0, 4.1) | 0 (0, 0.34) |
|  | 75 | 0 (0, 0) | 0 (0, 0) | 0 (0, 0) | 0 (0, 0) | 1.7 (0, 3.8) | 0 (0, 0.13) |
| Overweight - obesity | 5 | 11.6 (9.0, 14.2) | 9.2 (6.4, 11.9) | 4.8 (3.5, 6.2) | 8.1 (5.5, 10.7) | 5.8 (4.5, 7.1) | 9.6 (6.1, 13.1) |
|  | 15 | 8.8 (7.5, 10.2) | 5.3 (3.8, 6.8) | 3.9 (3.2, 4.6) | 5.0 (4.0, 6.0) | 4.8 (3.8, 5.7) | 3.7 (2.4, 5.0) |
|  | 25 | 5.4 (4.2, 6.6) | 2.7 (1.9, 3.5) | 2.9 (2.4, 3.5) | 2.7 (2.2, 3.1) | 3.5 (2.8, 4.3) | 1.1 (0, 1.7) |
|  | 35 | 2.8 (2.1, 3.4) | 1.2 (0, 1.7) | 2.1 (1.6, 2.6) | 1.3 (1.0, 1.6) | 2.4 (1.9, 2.9) | 0.37 (0.1, 0.64) |
|  | 45 | 0.94 (0.44, 1.4) | 0.43 (0, 0.81) | 1.4 (0, 1.9) | 0.61 (0.31, 0.9) | 1.6 (1.2, 2.0) | 0.18 (0, 0.51) |
|  | 55 | 0 (0, 0.27) | 0 (0, 0.31) | 0.87 (0.21, 1.5) | 0.21 (0, 0.6) | 0.99 (0, 1.7) | 0.21 (0, 0.74) |
|  | 65 | 0 (0, 0) | 0 (0, 0) | 0.42 (0, 1.2) | 0 (0, 0.34) | 0.54 (0, 1.5) | 0.28 (0, 1.1) |
|  | 75 | 0 (0, 0) | 0 (0, 0) | 0 (0, 0.74) | 0 (0, 0.29) | 0.19 (0, 1.2) | 0.21 (0, 1.4) |
